# LINC02709 facilitates hepatocellular carcinoma metastasis by increasing stem cell-like properties and suppressing Kupffer cell phagocytosis

**DOI:** 10.1038/s41420-026-03276-8

**Published:** 2026-08-12

**Authors:** Huamei Wei, Wenchuan Li, Xianjian Wu, Rongzhou Lu, Gaoxiong Tan, Yunyu Wei, Quan Fang, Youguan Huang, Zhongheng Wei, Jian Pu

**Affiliations:** 1 Department of Pathology, Affiliated Hospital of Youjiang Medical University for Nationalities, Baise, China; 2 Guangxi Clinical Medical Research Center of Hepatobiliary Disease, Baise, China; 3 Department of Hepatobiliary Surgery, Affiliated Hospital of Youjiang Medical University for Nationalities, Baise, China; 4 Graduate College of Youjiang Medical University for Nationalities, Baise, China

**Keywords:** Metastasis, Tumour immunology

## Abstract

Hepatocellular carcinoma (HCC) exhibits a high propensity for metastasis and recurrence, leading to poor prognosis. Intrahepatic metastasis is more common than extrahepatic spread. Metastases arise through diverse mechanisms, including the involvement of cancer stem cells (CSCs) and the immune-modulatory role of the liver tissue microenvironment. As hepatic resident macrophages, Kupffer cells play a critical role in immune surveillance by phagocytosing disseminated HCC cells. However, the molecules regulating HCC cell stemness properties, Kupffer cell phagocytosis, and further HCC intrahepatic metastasis remain poorly understood. In the present study, we found that the long noncoding RNA LINC02709 was upregulated in HCC and correlated with stemness properties, intrahepatic metastasis, and poor prognosis. LINC02709 not only enhanced stemness properties of HCC cells, but also suppressed the phagocytosis of HCC cells by Kupffer cells, leading to increased liver metastasis. LINC02709 upregulated CD24 and CD47, which mediated the effects of LINC02709 on HCC cell stemness properties and Kupffer cell phagocytosis. LINC02709 was identified as an m^6^A-modified transcript. m^6^A-modified LINC02709 bound and recruited the m^6^A reader FXR1 and the DNA demethylase TET1 to the *CD24* and *CD47* promoters, inducing DNA demethylation and transcriptional activation of *CD24* and *CD47*. The effects of LINC02709 on CD24/CD47, stemness properties, phagocytosis, and liver metastasis were all dependent on m^6^A modification. Our findings demonstrate that m^6^A-modified LINC02709 is a novel and promising biomarker for HCC recurrence and survival, enhancing HCC cell stemness properties, suppressing Kupffer cell phagocytosis, and driving HCC intrahepatic metastasis by epigenetically activating *CD24* and *CD47*. Targeting m^6^A-modified LINC02709 represents a promising therapeutic strategy for HCC.

## Introduction

Liver cancer ranks as the sixth-most common cancer globally, accounting for 4.3% of all cancer cases, yet it represents the third-leading cause of cancer-related deaths (7.8% of cancer mortality) [1]. Hepatocellular carcinoma (HCC) constitutes the predominant histological subtype of liver cancer [2]. While surgical resection may benefit early-diagnosed HCC patients, the majority eventually develop metastasis or post-surgery recurrence [3]. Of these cases, intrahepatic metastasis predominates over extrahepatic metastasis [4], highlighting the need to elucidate its molecular mechanisms to enable more effective therapeutic strategies [5].

Intrahepatic metastases arise through diverse mechanisms, including the involvement of cancer stem cells and the immune-modulatory role of the liver tissue microenvironment [6]. Accumulating evidences have confirmed that HCC cells with high stemness properties also show increased intrahepatic metastasis ability [7, 8]. The interaction between HCC cells and liver immune cells is another critical driver of intrahepatic metastasis [9–11]. Kupffer cells (KCs), the hepatic resident macrophages, represent the second-most abundant non-parenchymal cell population, outnumbered only by liver sinusoid endothelial cells [12–14]. Renowned for their phagocytic activity, KCs serve as key immune defenders against various tumor cells, including HCC cells [15–17]. However, to date, only ERMAP and ID3 have been identified as regulators of KC phagocytosis [18, 19], leaving the roles of other molecules in KC phagocytosis and HCC intrahepatic metastasis largely unexplored.

Mounting evidences highlight the critical roles of epigenetic modifications in various pathophysiological processes, including tumorigenesis and immunity [20–22]. Many oncogenes and tumor suppressors are regulated by epigenetic modifications, including chromatin remodeling (DNA methylation, histone acetylation), noncoding RNAs (microRNAs, long noncoding RNAs), and RNA modifications (N^6^-methyladenosine, N^1^-methyladenosine, and N^4^-acetylcytosine) [23–30]. DNA methylation (5-methylcytosine) typically suppresses gene expression [31, 32]. Long noncoding RNAs (lncRNAs) (>200 nucleotides with limited coding potential) show aberrant expression and diverse roles in many diseases [33–40]. However, their involvement in KC phagocytosis remains largely unexplored. Among RNA modifications, N^6^-methyladenosine (m^6^A) is the most prevalent RNA modification and implicated in the initiation and progression of many diseases through modulating RNA stability, translation, and function [41–48], yet its role in KC phagocytosis and HCC metastasis remains unclear.

In this study, through analysis of The Cancer Genome Atlas (TCGA) Liver Hepatocellular Carcinoma (LIHC) dataset, we identified LINC02709 as a stemness- and recurrence- associated lncRNA in HCC. LINC020709 promotes HCC intrahepatic metastasis through m^6^A-dependent increasing of stemness properties and suppression of KC phagocytosis. Specifically, m^6^A-modified LINC02709 recruits the m^6^A reader FXR1 and the DNA demethylase TET1 to the *CD24* and *CD47* promoters, inducing DNA hypomethylation and subsequent transcriptional activation of *CD24* and *CD47*. Functional rescue experiments established CD24 and CD47 as key effectors mediating LINC02709’s roles in promoting HCC cell stemness properties, inhibiting KC phagocytosis, and driving HCC metastasis.

## Results

### LINC02709 is associated with stemness properties and intrahepatic metastasis

**Fig. 1:**
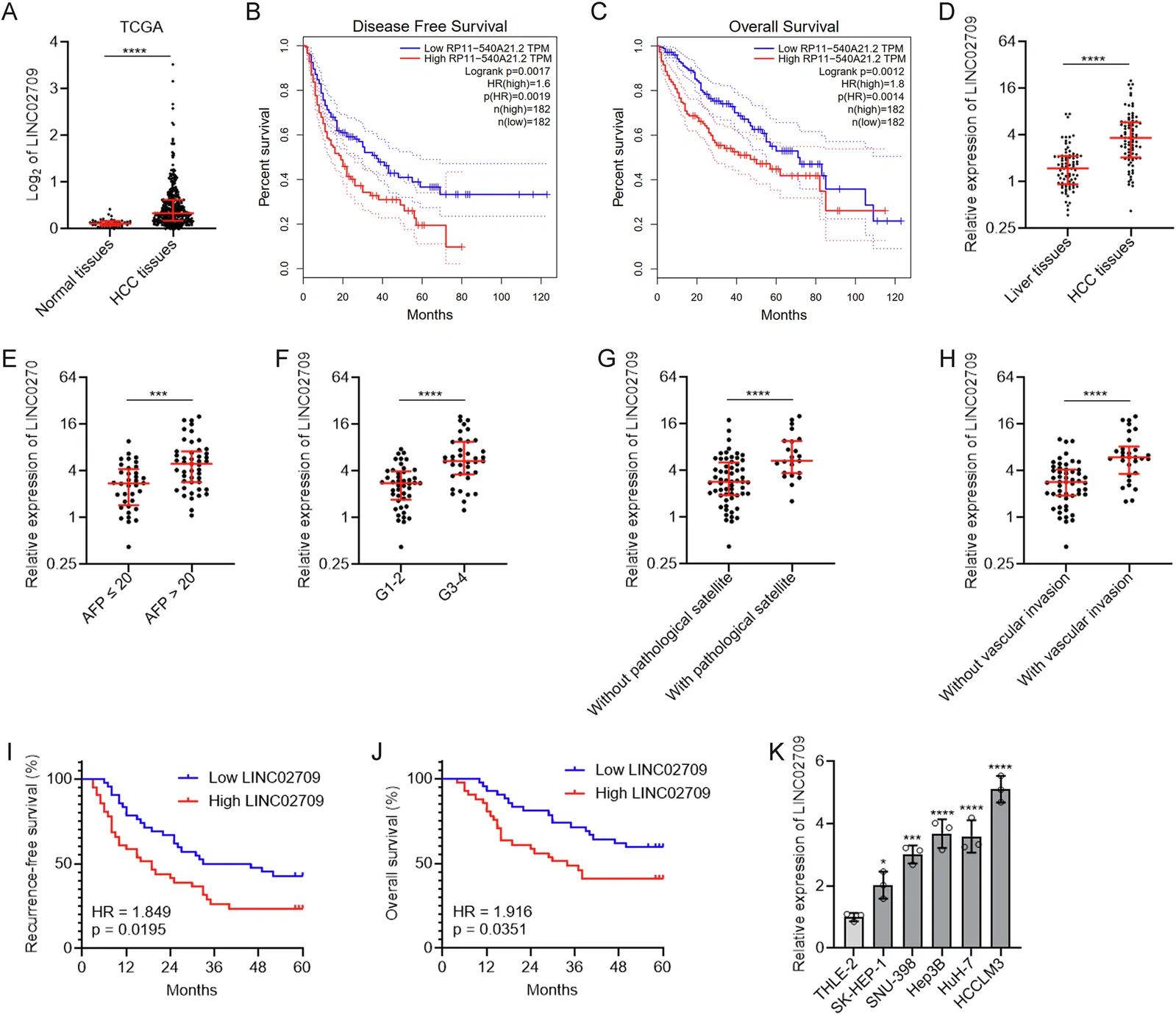
LINC02709 is associated with stemness properties, intrahepatic metastasis, and poor prognosis in HCC. **A** LINC02709 expression in 371 HCC tissues versus 50 normal liver tissues from TCGA LIHC dataset. GEPIA analysis of the correlation between LINC02709 expression (annotated as RP11-540A21.2 in TCGA) and disease-free survival (**B**) and overall survival (**C**) derived from TCGA LIHC dataset. **D** qPCR analysis of LINC02709 expression in 83 pairs HCC and matched adjacent noncancerous tissues. **E** qPCR analysis of LINC02709 expression in 36 HCC tissues with AFP value ≤ 20 and 47 HCC tissues with AFP value > 20. **F** qPCR analysis of LINC02709 expression in 45 HCC tissues with histologic grade 1-2 and 38 HCC tissues with histologic grade 3-4. **G** qPCR analysis of LINC02709 expression in 60 HCC tissues without pathological satellites and 23 HCC tissues with pathological satellites. **H** qPCR analysis of LINC02709 expression in 53 HCC tissues without vascular invasion and 30 HCC tissues with vascular invasion. **I**, **J** Kaplan–Meier survival analysis showing LINC02709’s association with recurrence-free survival (**I**; HR = 1.849, *p* = 0.0195) and overall survival (**J**; HR = 1.916, *p* = 0.0351) in our HCC cohort (*n* = 83). **K** qPCR analysis of LINC02709 expression in immortalized liver cell line THLE-2 and HCC cell lines SK-HEP-1, SNU-398, Hep3B, HuH-7, and HCCLM3. For (**A**) and (**D**–**H**), data are presented as median with interquartile range. For (**K**), data are presented as mean ± standard deviation (SD) of *n* = 3 biological replicates. *p < 0.01, ***p < 0.001, ****p < 0.0001 by Mann–Whitney test (**A**, **E**–**H**), Wilcoxon matched-pairs signed rank test (**D**), log-rank test (**I**, **J**), or one-way ANOVA with Dunnett’s multiple comparisons test (**K**).

To investigate the expression of LINC02709 in HCC, we analyzed TCGA LIHC RNA-seq data and observed significant upregulation of LINC02709 in HCC tissues (Fig. 1A). Correlation analyses further revealed positive associations between LINC02709 expression and clinicopathological features, including α-fetoprotein (AFP) levels, tumor grade, pathologic T stage, vascular invasion, and TNM stage (Table 1). GEPIA analysis demonstrated that high LINC02709 expression correlated with poor disease-free and overall survival (Fig. 1B, C). Validation in our cohort of 83 paired HCC and adjacent non-tumor liver tissues confirmed elevated LINC02709 expression in HCC (Fig. 1D). Notably, LINC02709 levels were further increased in HCC tissues with high AFP (Fig. 1E), poor differentiation (Fig. 1F), pathological satellite (Fig. 1G), and vascular invasion (Fig. 1H). Kaplan–Meier analyses in our HCC cohort reinforced the prognostic significance of LINC02709, linking high expression to reduced recurrence-free and overall survival (Fig. 1I, J). Moreover, LINC02709 was upregulated in HCC cell lines (SK-HEP-1, SNU-398, Hep3B, HuH-7, and LM3) compared to the immortalized liver cell line THLE-2 (Fig. 1K). *LINC02709*, located at chr11p15.4, consists of three exons and 2311 nucleotides. Together, these findings suggested that LINC02709 correlates with stemness properties, intrahepatic metastasis, and recurrence of HCC.

**Table 1:** Correlation between LINC02709 expression levels and clinicopathological characteristics of HCC patients according to TCGA dataset.

Feature	LINC02709 expression		χ^2^	*p*-value^*^
	Low	High^a^		
Age			0.260	0.610
>50	144	148		
≤50	41	37		
Gender			2.881	0.090
Male	133	117		
Female	53	68		
Child-Pugh			1.396	0.498
A	108	109		
B	12	9		
C	1	0		
AFP (ng/ml)			4.117	0.042
>20	58	73		
≤20	83	64		
Grade			23.128	0.000
G1	41	14		
G2	93	84		
G3	47	75		
G4	3	9		
Tumor pathologic pt			12.303	0.006
T1	106	75		
T2	35	59		
T3	36	44		
T4	6	7		
Vascular invasion			8.925	0.012
None	115	91		
Micro	37	56		
Macro	5	11		
TNM staging			11.664	0.009
I	99	72		
II	33	53		
III	38	47		
IV	4	1		

*p-value was acquired by Pearson chi-square tests.

^a^The medium expression level (0.3391) was used as the cutoff.

### LINC02709 enhances stemness properties and intrahepatic metastasis of HCC

**Fig. 2:**
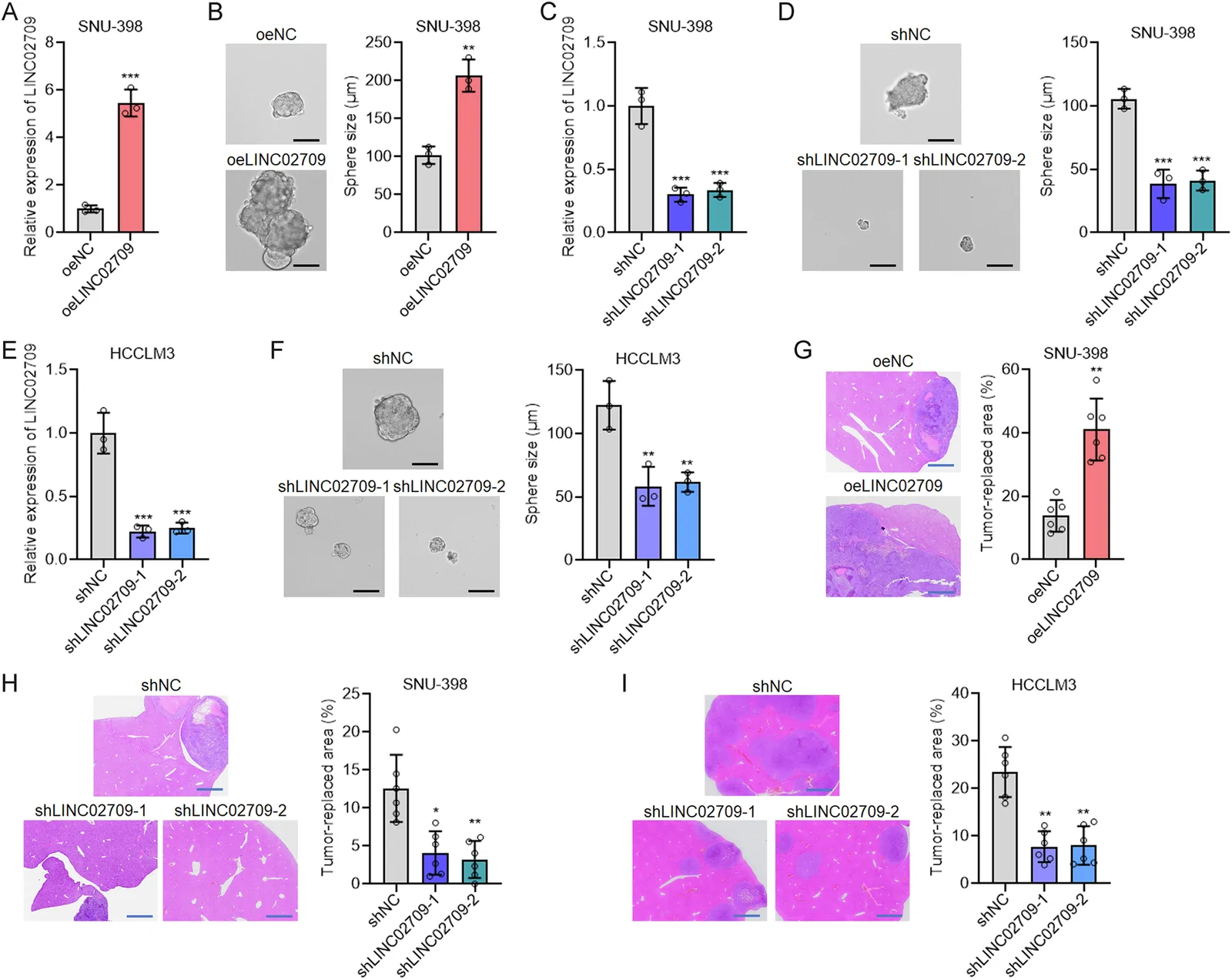
LINC02709 enhances stemness properties and liver metastasis of HCC cells. **A** qPCR analysis of LINC02709 expression in SNU-398 cells with stable LINC02709 overexpression. **B** The spheroids form ability of SNU-398 cells with stable LINC02709 overexpression. Scale bars = 100 μm. **C** qPCR analysis of LINC02709 knockdown efficiency in SNU-398 cells. **D** The spheroids form ability of SNU-398 cells with stable LINC02709 knockdown. Scale bars = 100 μm. **E** qPCR analysis of LINC02709 knockdown efficiency in HCCLM3 cells. **F** The spheroids form ability of HCCLM3 cells with stable LINC02709 knockdown. Scale bars = 100 μm. Liver metastasis assessed by H&E staining at day 49 after intrasplenic injection of 5 × 10^6^ SNU-398 cells with LINC02709 overexpression (**G**) or knockdown (**H**). Scale bars = 1 mm. **I** Liver metastasis assessed by H&E staining at day 35 after intrasplenic injection of 5 × 10^6^ HCCLM3 cells with LINC02709 knockdown. Scale bars = 1 mm. Data are presented as mean ± SD of *n* = 3 biological replicates (**A**–**F**) or *n* = 6 mice per group (**G**–**I**). *p < 0.05, **p < 0.01, ***p < 0.001 by Student’s *t* test (**A**, **B**), one-way ANOVA with Dunnett’s multiple comparisons test (**C**–**F**), Mann–Whitney test (**G**), or Kruskal–Wallis test with Dunn’s multiple comparisons test (**H**, **I**).

Due to the correlation between LINC02709 expression and AFP and tumor grade, we investigated whether LINC02709 modulates stemness properties of HCC.We generated LINC02709-overexpressing SNU-398 cells (Fig. 2A). Sphere formation assays revealed that overexpression of LINC02709 enhanced the stemness properties of SNU-398 cells (Fig. 2B). Conversely, LINC02709 knockdown decreased the stemness properties of SNU-398 cells (Fig. 2C, D). Similar results were observed in HCCLM3 cells, where LINC02709 knockdown also decreased the stemness properties of HCCLM3 cells (Fig. 2E, F). Due to the correlation between LINC02709 expression and pathological satellite and vascular invasion, we further investigated the potential roles of LINC02709 in intrahepatic metastasis. In vivo liver metastasis assays revealed that LINC02709-overexpressed SNU-398 cells formed more liver metastatic lesions than controls (Fig. 2G). Conversely, LINC02709-depleted SNU-398 cells exhibited reduced liver metastatic potential (Fig. 2H). Similar results were observed in HCCLM3 cells, where LINC02709 knockdown also suppressed liver metastasis in nude mice (Fig. 2I). These findings demonstrate that LINC02709 enhances stemness properties and facilitates liver metastasis of HCC cells.

### LINC02709 inhibits phagocytosis of HCC cells by Kupffer cells

**Fig. 3:**
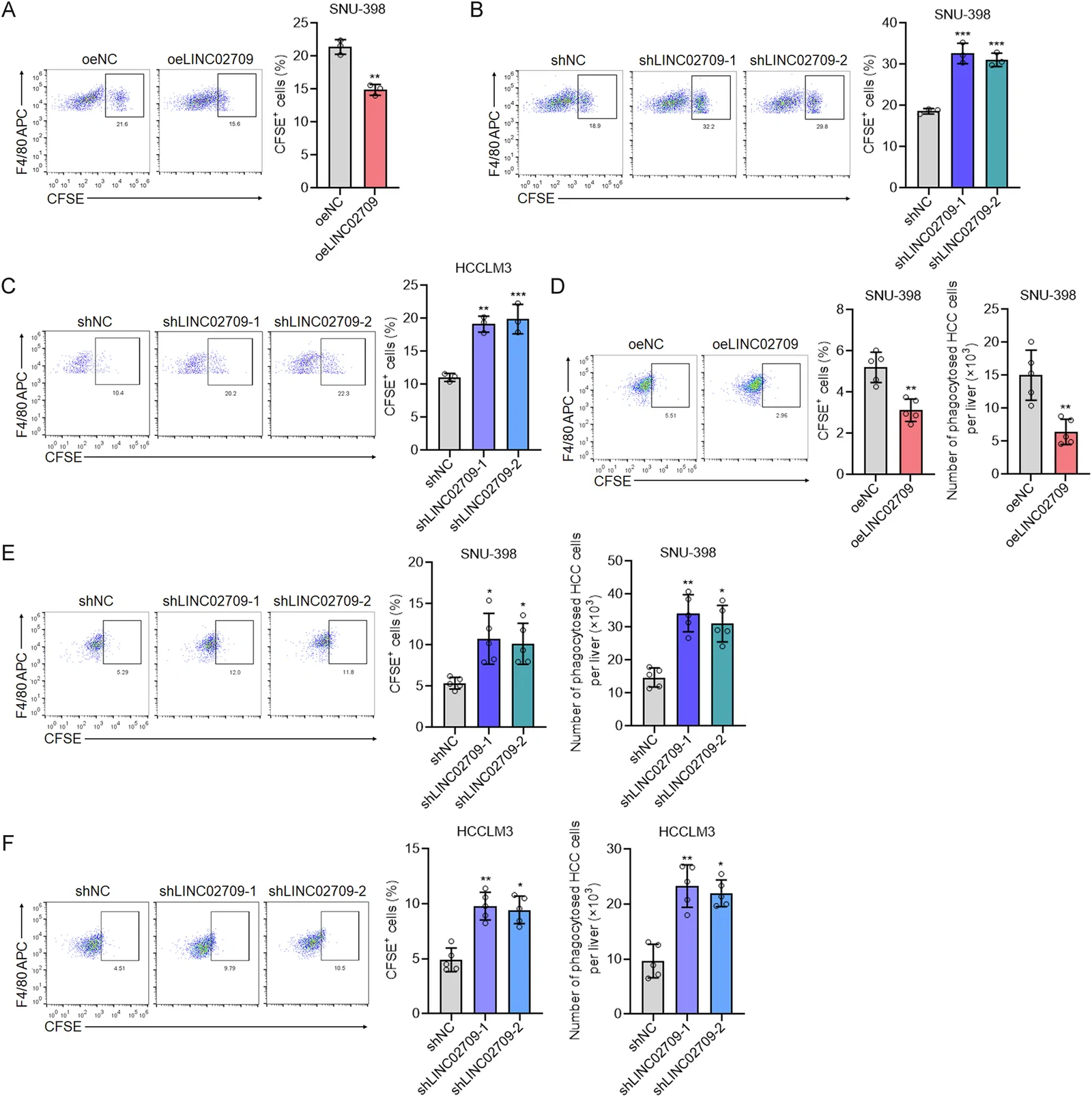
LINC02709 inhibits the phagocytosis of HCC cells by Kupffer cells. Flow cytometry analysis of in vitro phagocytosis of CFSE-labeled SNU-398 cells with LINC02709 overexpression (**A**) or knockdown (**B**) by KCs. **C** Flow cytometry analysis of in vitro phagocytosis of CFSE-labeled HCCLM3 cells with LINC02709 knockdown by KCs. Flow cytometry analysis of in vivo phagocytosis of CFSE-labeled SNU-398 cells with LINC02709 overexpression (**D**) or knockdown (**E**) by KCs 12 h post-intrasplenic inoculation. **F** Flow cytometry analysis of in vivo phagocytosis of CFSE-labeled HCCLM3 cells with LINC02709 knockdown by KCs 12 h post-intrasplenic inoculation. Data are presented as mean ± SD of *n* = 3 biological replicates (**A**–**C**) or *n* = 5 mice per group (**D**–**F**). *p < 0.05, **p < 0.01, ***p < 0.001 by Student’s *t* test (**A**), one-way ANOVA with Dunnett’s multiple comparisons test (**B**, **C**), Mann–Whitney test (**D**), or Kruskal–Wallis test with Dunn’s multiple comparisons test (**E**, **F**).

Kupffer cells (KCs), the liver-resident macrophages, are the key immune defenders against tumor cells and protect the liver from tumor liver metastasis. To elucidate whether KCs mediates the roles of LINC02709 in HCC intrahepatic metastasis, we next detected whether LINC02709 regulates the phagocytosis of HCC cells by KCs, CFSE-labeled HCC cells were co-cultured with KCs, and flow cytometry revealed that LINC02709 overexpression in SNU-398 cells decreased CFSE intensity in KCs (Fig. 3A), indicating reduced in vitro phagocytosis. This effect was confirmed in LINC02709-overexpressing SK-HEP-1 cells (Supplementary Fig. S1A, B). Conversely, LINC02709 depletion in SNU-398 or HCCLM3 cells enhanced in vitro phagocytosis of these cells by KCs (Fig. 3B, C). We further validated these findings in vivo. CFSE-labeled HCC cells were intrasplenically injected into nude mice, and 12 h later, in vivo phagocytosis of HCC cells by KCs was measured using flow cytometry. The results demonstrated that LINC02709 overexpression reduced in vivo phagocytosis of SNU-398 and SK-HEP-1 cells by KCs (Fig. 3D and Supplementary Fig. S1C), while LINC02709 knockdown promoted in vivo phagocytosis of SNU-398 and HCCLM3 cells by KCs (Fig. 3E, F). Together, these results demonstrate that LINC02709 suppresses the phagocytosis of HCC cells by KCs.

### LINC02709 upregulates CD47 and CD24 expression

**Fig. 4:**
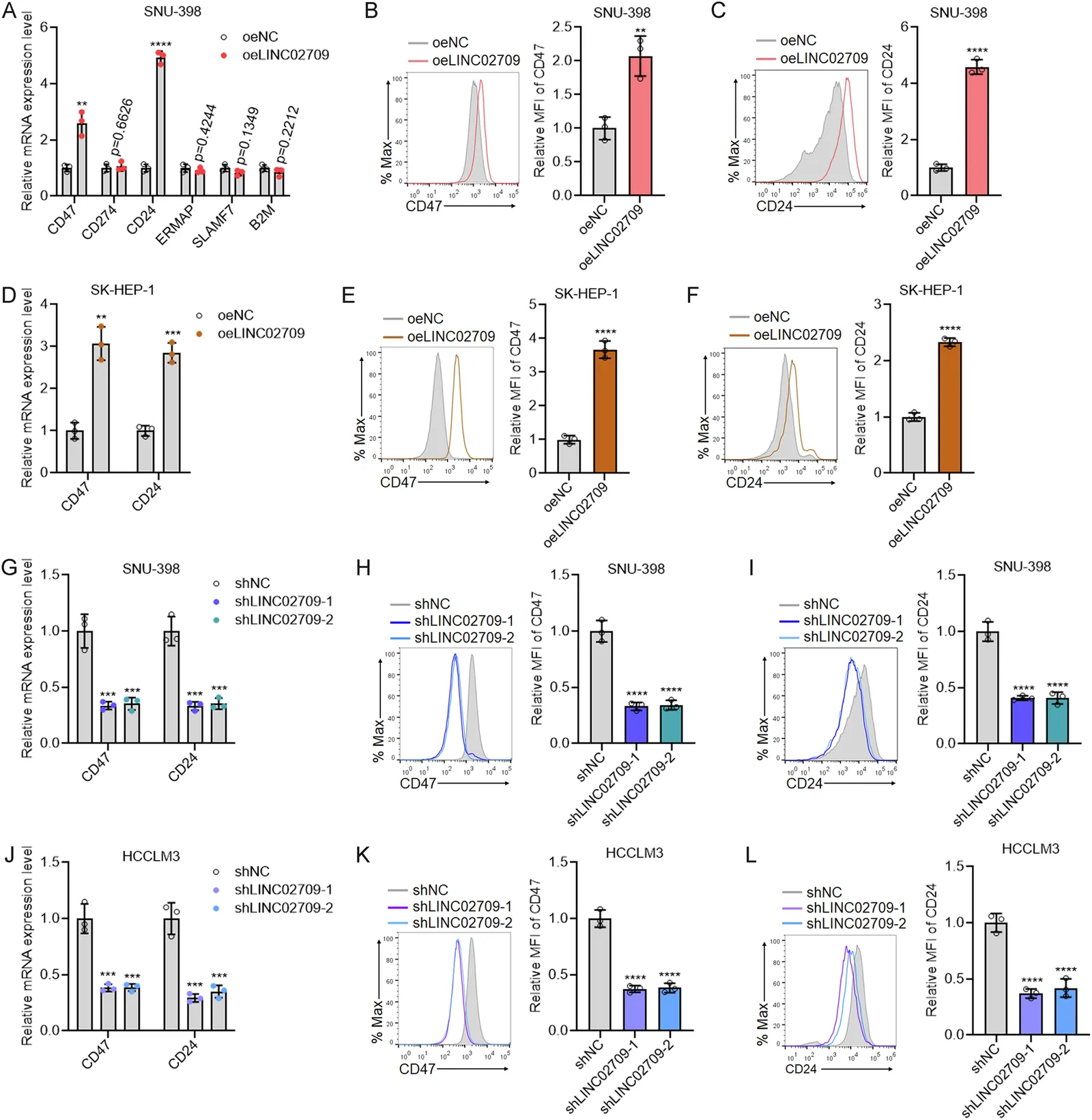
LINC02709 upregulates CD47 and CD24 expression in HCC cells. **A** qPCR analysis of mRNA levels of phagocytosis checkpoints in SNU-398 cells with LINC02709 overexpression. Flow cytometry quantification of CD47 (**B**) or CD24 (**C**) protein levels in SNU-398 cells with LINC02709 overexpression. **D** qPCR analysis of CD47 and CD24 mRNA levels in SK-HEP-1 cells with LINC02709 overexpression. Flow cytometry quantification of CD47 (**E**) or CD24 (**F**) protein levels in SK-HEP-1 cells with LINC02709 overexpression. **G** qPCR analysis of CD47 and CD24 mRNA levels in SNU-398 cells with LINC02709 knockdown or control. Flow cytometry quantification of CD47 (**H**) or CD24 (**I**) protein levels in SNU-398 cells with LINC02709 knockdown or control. **J** qPCR analysis of CD47 and CD24 mRNA levels in HCCLM3 cells with LINC02709 knockdown or control. Flow cytometry quantification of CD47 (**K**) or CD24 (**L**) protein levels in HCCLM3 cells with LINC02709 knockdown or control. Data are presented as mean ± SD of *n* = 3 biological replicates. **p < 0.01, ***p < 0.001, ****p < 0.0001, ns not significant, by Student’s *t* test (**A**–**F**) or one-way ANOVA wi*t*h Dunnett’s multiple comparisons test (**G**–**L**).

Subcellular localization assays identified LINC02709 as a chromatin-bound RNA (Supplementary Fig. S2). Thus, we examined its potential role in regulating the transcription of key phagocytosis checkpoint genes *CD47*, *CD274* (encoding PD-L1), *CD24*, *ERMAP*, *SLAMF7*, and *B2M* (encoding β2-microglobulin). In SNU-398 cells, LINC02709 overexpression significantly upregulated *CD47* and *CD24* mRNA levels (Fig. 4A), with corresponding increases in CD47 and CD24 protein expression confirmed by flow cytometry (Fig. 4B, C). This effect was reproducible in SK-HEP-1 cells, where LINC02709 overexpression similarly elevated *CD47* and *CD24* transcripts and protein levels (Fig. 4D–F). Conversely, LINC02709 depletion in SNU-398 cells reduced both *CD47* and *CD24* mRNA and protein expression (Fig. 4G–I). Consistent with these findings, knockdown of LINC02709 in HCCLM3 cells also decreased *CD47* and *CD24* expression at the mRNA and protein levels (Fig. 4J–L). These results demonstrate that chromatin-associated LINC02709 transcriptionally upregulates CD47 and CD24, two critical immune checkpoint molecules involved in phagocytosis evasion. Intriguingly, CD24 is also a key liver cancer stem cell marker and enhances stemness properties of HCC cells [51, 52].

### CD47 and CD24 mediates the effects of LINC02709 on Kupffer cell phagocytosis and stemness properties of HCC cells

**Fig. 5:**
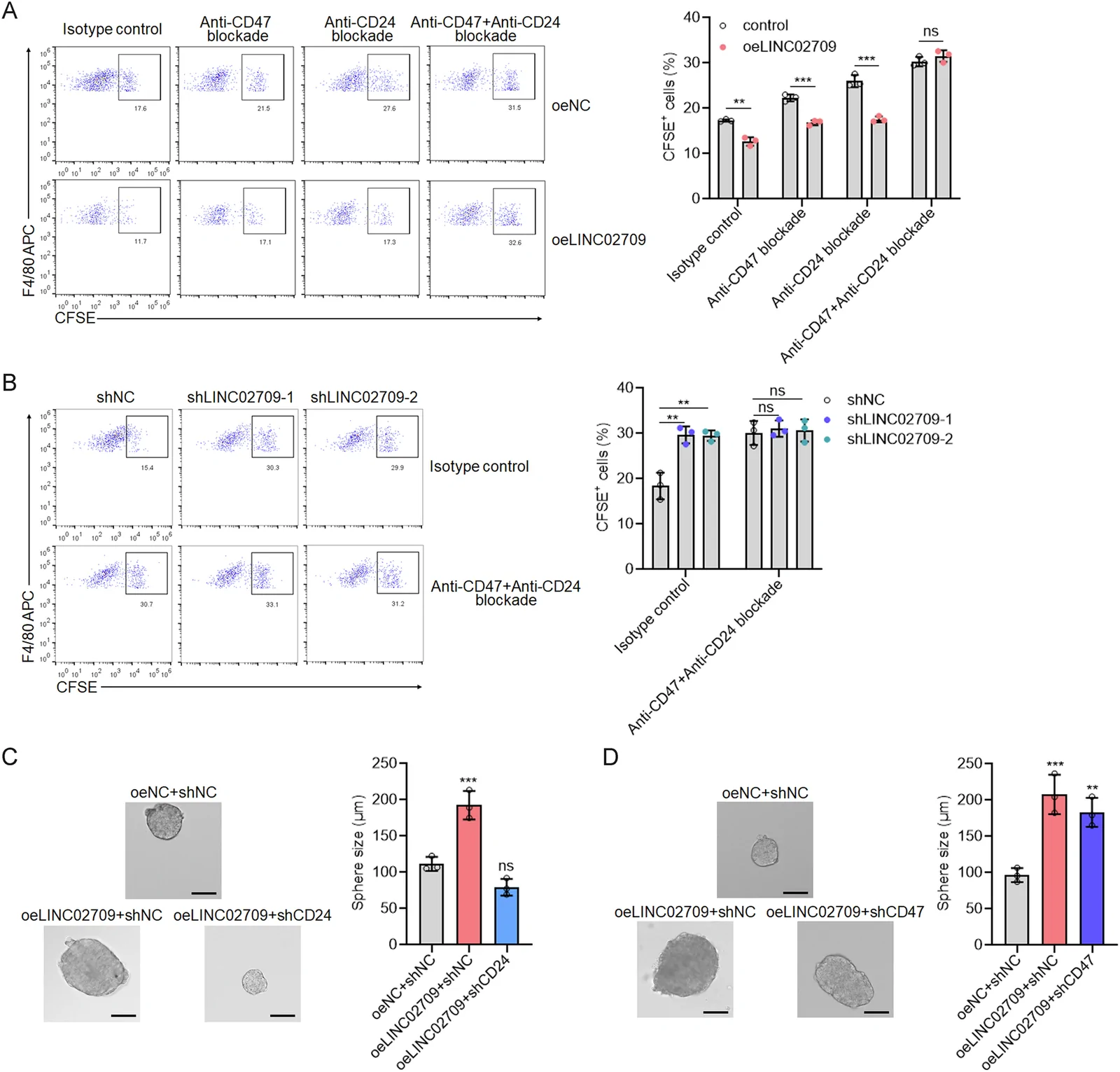
CD47 and CD24 mediates the effects of LINC02709 on Kupffer cell phagocytosis and stemness properties of HCC cells. **A** Flow cytometry analysis of phagocytosis of CFSE-labeled SNU-398 cells with LINC02709 overexpression by KCs, with or without CD47, CD24, or combined CD47/CD24 neutralizing antibodies. **B** Flow cytometry analysis of phagocytosis of CFSE-labeled SNU-398 cells with LINC02709 knockdown by KCs treated with CD47 and CD24 neutralizing antibodies. **C** The spheroids form ability of SNU-398 cells with LINC02709 overexpression and CD24 knockdown. Scale bars = 100 μm. **D** The spheroids form ability of SNU-398 cells with LINC02709 overexpression and CD47 knockdown. Scale bars = 100 μm. Data are presented as mean ± SD of *n* = 3 biological replicates. **p < 0.01, ***p < 0.001, ns not significant, by Student’s *t* test (**A**) or one-way ANOVA with Dunnett’s multiple comparisons test (**B**, **C**).

To determine whether CD47 and CD24 mediate LINC02709’s effects on KC phagocytosis, we performed antibody blockade experiments. Neutralizing antibodies against CD47 or CD24 enhanced KC phagocytosis (Fig. 5A). Only dual blockade of CD47 and CD24 completely abolished the suppressive effect of LINC02709 on phagocytosis (Fig. 5A). Similarly, the increased phagocytosis observed in LINC02709-depleted cells was fully reversed by combined CD47 and CD24 inhibition (Fig. 5B). To determine whether CD24 and CD47 mediate LINC02709’s effects on stemness properties of HCC cells, we depleted CD24 or CD47 in LINC02709-overexpressing SNU-398 cells, and the sphere formation assays revealed that only CD24 knockdown reversed the increased stemness properties of SNU-398 cells (Fig. 5C), and while CD47 knockdown showed no notable effect on the stemness properties of SNU-398 cells (Fig. 5D). These results demonstrate that LINC02709 modulates KC phagocytosis through a cooperative mechanism requiring both CD47 and CD24. LINC02709 enhances stemness properties of HCC cells through upregulating CD24.

### LINC02709 activates CD47 and CD24 transcription via m^6^A-dependent DNA demethylation

**Fig. 6:**
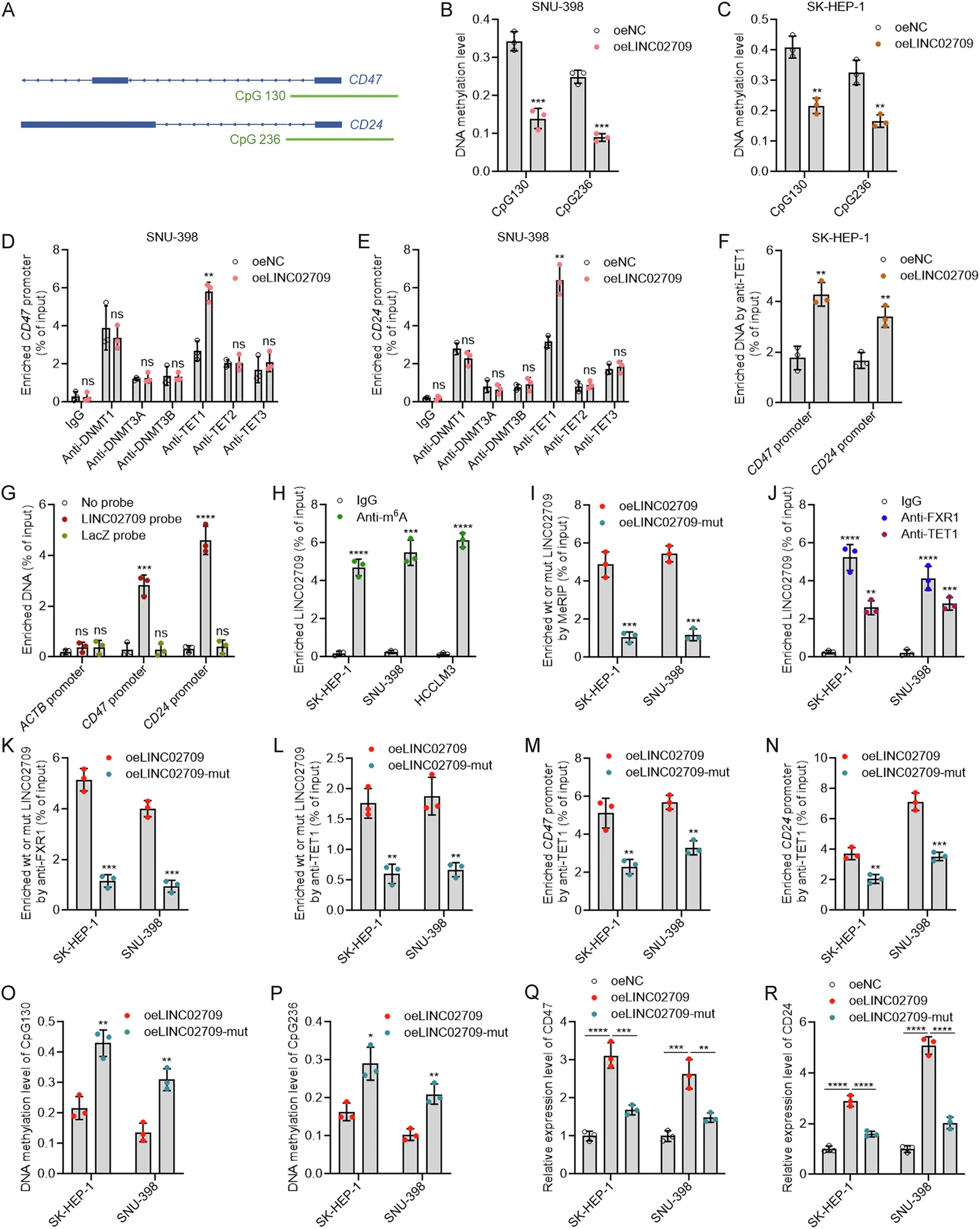
LINC02709 recruits FXR1 and TET1 to *CD47*/*CD24* promoters, leading to DNA demethylation and transcriptional activation of *CD47*/*CD24* in an m^6^A-dependent manner. **A** Genomic loci of *CD47* and *CD24* (human genome browser hg38 version), with green boxes indicating CpG islands. Bisulfate sequencing of *CD47*/*CD24* promoters’ methylation in SNU-398 (**B**) or SK-HEP-1 (**C**) cells with LINC02709 overexpression or control. CUT&RUN analysis of DNMT1, DNMT3A, DNMT3B, TET1, TET2, and TET3 binding to *CD47* (**D**) or *CD24* (**E**) promoters in LINC02709-overexpressing SNU-398 cells. **F** CUT&RUN analysis of TET1 binding to *CD47*/*CD24* promoters in SK-HEP-1 cells with LINC02709 overexpression. **G** ChIRP analysis of the binding of LINC02709 to *CD47*/*CD24* promoters in SNU-398 cells. LacZ probe served as negative control probe. **H** MeRIP analysis of m^6^A modification on endogenous LINC02709 in SK-HEP-1, SNU-398, and HCCLM3 cells. **I** MeRIP analysis of m^6^A modification on wild-type or mutant (854/1172/1460 sites) LINC02709 in SK-HEP-1 and SNU-398 cells. **J** RIP analysis of the binding of FXR1 and TET1 to LINC02709 in SK-HEP-1 and SNU-398 cells. RIP analysis of the binding of FXR1 (**K**) or TET1 (**L**) to wild-type or mutant LINC02709. CUT&RUN analysis of the binding of TET1 to *CD47* (**M**) or *CD24* (**N**) promoters in SK-HEP-1 and SNU-398 cells expressing wild-type or mutant LINC02709. Bisulfate sequencing of *CD47* (**O**) or *CD24* (**P**) promoter methylation in SK-HEP-1 and SNU-398 cells expressing wild-type or mutant LINC02709. qPCR analysis of *CD47* (**Q**) or *CD24* (**R**) expression in SK-HEP-1 and SNU-398 cells expressing wild-type or mutant LINC02709. Data are presented as mean ± SD of *n* = 3 biological replicates. *p < 0.05, **p < 0.01, ***p < 0.001, ****p < 0.0001, ns not significant, by Student’s *t* test (**B**–**F**, **H**, **I**, **K**–**P**) or one-way ANOVA wi*t*h Dunnett’s multiple comparisons test (**G**, **J**, **Q**, **R**).

As a chromatin-associated RNA, LINC02709 upregulates the expression of *CD47* and *CD24*. Promoter analysis revealed that both *CD47* and *CD24* promoters are located within CpG islands (Fig. 6A). Bisulfite sequencing demonstrated that LINC02709 overexpression significantly reduced DNA methylation at the *CD47*/*CD24* promoters in SNU-398 and SK-HEP-1 cells (Fig. 6B, C), while LINC02709 depletion increased methylation levels in SNU-398 cells and HCCLM3 cells (Supplementary Fig. S3A, B). To investigate whether LINC02709 influences the recruitment of DNA methyltransferases and demethylases to the *CD47*/*CD24* promoters, we performed CUT&RUN assays. In SNU-398 cells, LINC02709 overexpression selectively enhanced TET1 recruitment to the *CD47*/*CD24* promoters, but had no detectable effect on DNMT1, DNMT3A, DNMT3B, TET2, or TET3 recruitment (Fig. 6D, E). LINC02709 did not affect TET1 recruitment to the *CD274* and *ERMAP* promoters (Supplementary Fig. S3C), supporting the specificity of this recruitment mechanism. A similar *CD47*/*CD24* promoters-specific TET1-recruitment effect was observed in SK-HEP-1 cells (Fig. 6F and Supplementary Fig. S3D), whereas LINC02709 knockdown reduced TET1 recruitment in SNU-398 cells and HCCLM3 cells (Supplementary Fig. S3E, F). Prior studies, including our own, have established that m^6^A-modified RNAs interact with the m^6^A reader FXR1, which subsequently recruits TET1 to target gene promoters, modulating DNA methylation and transcriptional activity [44, 53]. To test whether LINC02709 employs this mechanism, we performed ChIRP assays, confirming its direct binding to the *CD47*/*CD24* promoters (Fig. 6G), but no binding to *CD274*/*ERMAP* promoters (Supplementary Fig. S3G). Using the online in silico tool SRAMP (http://www.cuilab.cn/sramp), we predicted three m^6^A modification sites on LINC02709 (Supplementary Fig. S3H), which were experimentally validated by MeRIP assays in SK-HEP-1, SNU-398, and HCCLM3 cells (Fig. 6H). To verify the m^6^A modification sites and assess their functional relevance, we generated a triple m^6^A site-mutant LINC02709 plasmid. MeRIP assays confirmed that these mutations nearly abolished m^6^A modification (Fig. 6I), confirming these sites as the primary m^6^A residues. RIP assays revealed that LINC02709 binds FXR1 and TET1 in SK-HEP-1 and SNU-398 cells (Fig. 6J), an interaction that was strongly diminished by m^6^A sites mutations (Fig. 6K, L). Accordingly, CUT&RUN assays showed that LINC02709-mediated TET1 recruitment to *CD47*/*CD24* promoters was largely eliminated upon m^6^A sites disruption (Fig. 6M, N). Bisulfite DNA sequencing showed that the reduction in *CD47*/*CD24* promoter DNA methylation induced by LINC02709 overexpression was also dependent on intact m^6^A sites (Fig. 6O, P). Similarly, upregulation of *CD47* and *CD24* induced by LINC02709 overexpression was also dependent on intact m^6^A sites (Fig. 6Q, R). To determine whether m^6^A-modified LINC02709 regulates *CD47*/*CD24* expression in an FXR1/TET1-dependent manner, we depleted FXR1 or TET1 in LINC02709-overexpressing SNU-398 cells. qPCR analysis revealed that depletion of either FXR1 or TET1 largely abolished the upregulation of *CD47* and *CD24* induced by LINC02709 overexpression (Supplementary Fig. S3I, J).

**Fig. 7:**
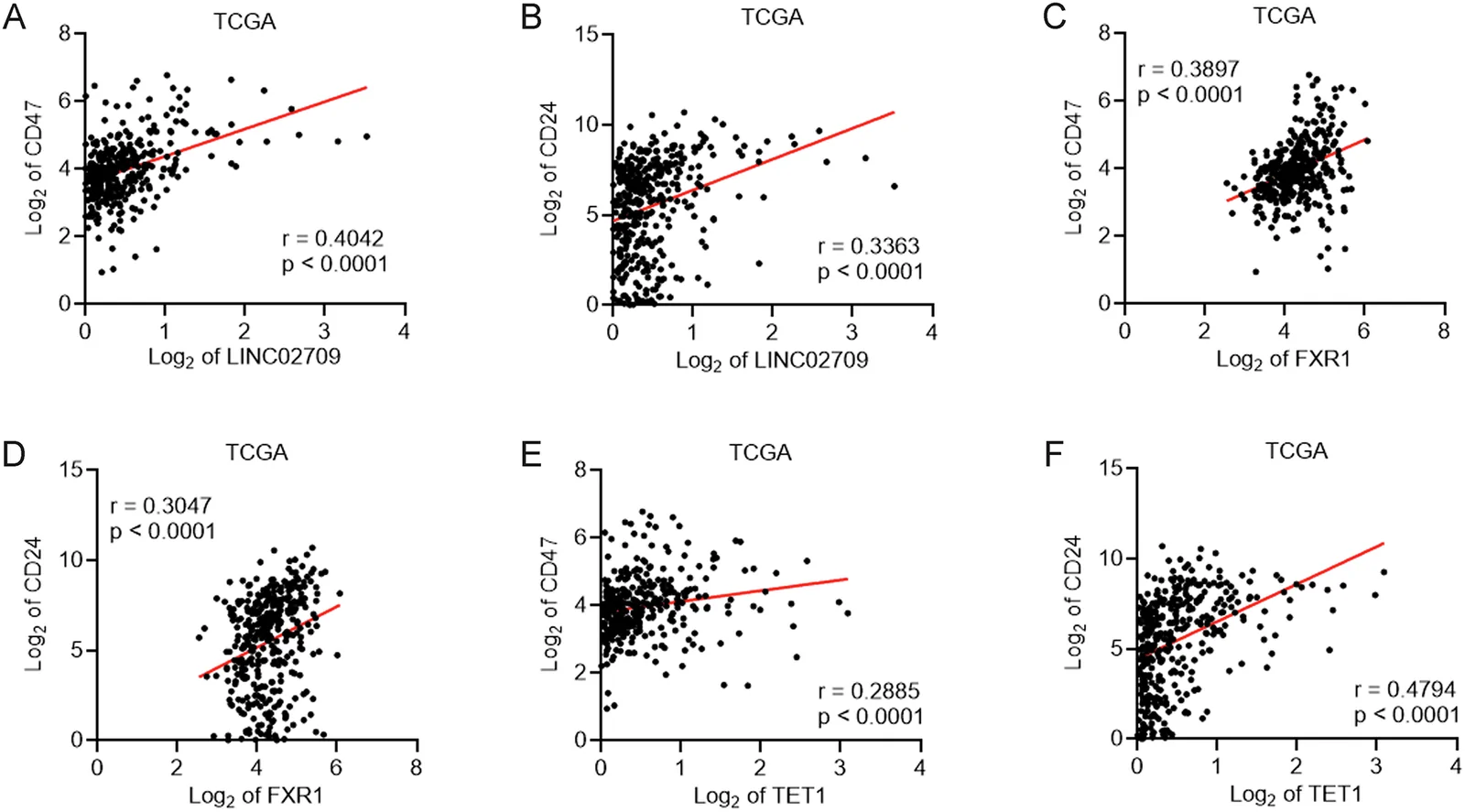
Clinical correlations between LINC02709, FXR1, TET1 and CD47, CD24 expression in HCC tissues. Clinical correlations of LINC02709 expression with CD47 (**A**) or CD24 (**B**) expression in 371 HCC tissues derived from TCGA LIHC dataset. Clinical correlations of FXR1 expression with CD47 (**C**) or CD24 (**D**) expression in 371 HCC tissues derived from TCGA LIHC dataset. Clinical correlations of TET1 expression with CD47 (**E**) or CD24 (**F**) expression in 371 HCC tissues derived from TCGA LIHC dataset. r and *p* values were calculated by Spearman correlation analysis.

Supporting these findings, TCGA LIHC data revealed significant positive correlations between LINC02709 and CD47/CD24 expression in HCC tissues (Fig. 7A, B), FXR1 and CD47/CD24 expression in HCC tissues (Fig. 7C, D), TET1 and CD47/CD24 expression in HCC tissues (Fig. 7E, F), supporting the clinical relevance of LINC02709/FXR1/TET1/CD47/CD24 regulatory axis.

### LINC02709’ effects on HCC stemness properties, Kupffer cell phagocytosis, and liver metastasis are m^6^A-dependent

**Fig. 8:**
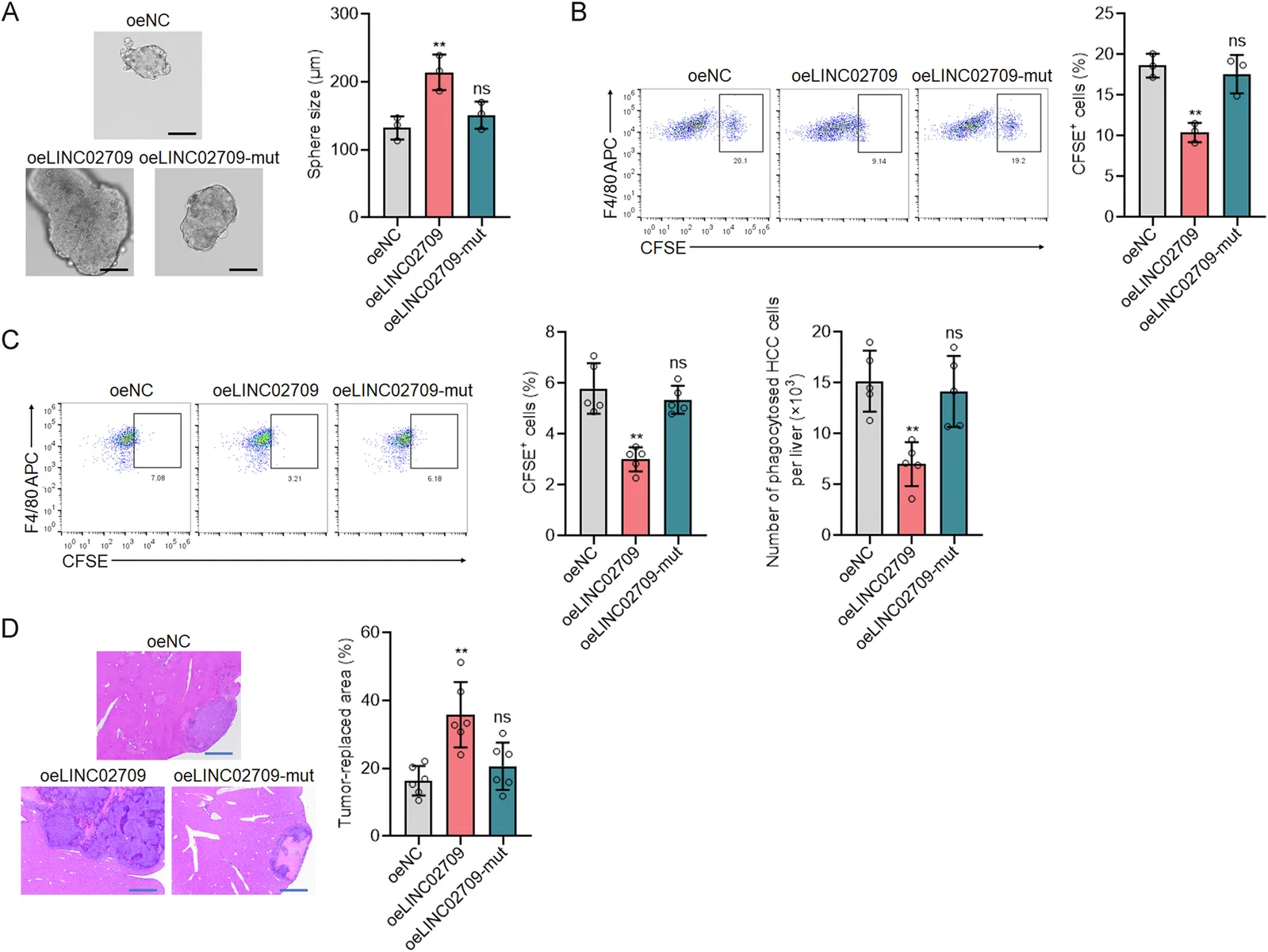
m^6^A modification of LINC02709 mediates the increasing of stemness properties, suppression of Kupffer cell phagocytosis, and promotion of liver metastasis. **A** The spheroids form ability of SNU-398 cells overexpressing wild-type or mutantLINC02709. Scale bars = 100 μm. **B** Flow cytometry analysis of in vitro phagocytosis of CFSE-labeled SNU-398 cells overexpressing wild-type or mutant LINC02709 by KCs. **C** Flow cytometry analysis of in vivo phagocytosis of CFSE-labeled SNU-398 cells overexpressing wild-type or mutant LINC02709 by KCs 12 h post-intrasplenic inoculation. **D** Liver metastasis assessed by H&E staining at day 49 after intrasplenic injection of 5 × 10^6^ SNU-398 cells with wild-type or mutant LINC02709 overexpression. Scale bars = 1 mm. Data are presented as mean ± SD of *n* = 3 biological replicates (**A**, **B**), *n* = 5 mice per group (**C**), or *n* = 6 mice per group (**D**). **p < 0.01, ns not significant, by one-way ANOVA with Dunnett’s multiple comparisons test (**A**, **B**) or Kruskal–Wallis test with Dunn’s multiple comparisons test (**C**, **D**).

To determine whether LINC02709’s biological functions depend on m^6^A modification, we performed sphere formation assays using SNU-398 cells stably overexpressing either wild-type LINC02709 or m^6^A sites mutant. The results showed that mutations in the m^6^A modification sites abrogated LINC02709’s effect on HCC cells stemness properties (Fig. 8A). In vitro and in vivo phagocytosis assays showed that mutations in the m^6^A modification sites abrogated LINC02709’s effect on KC phagocytosis (Fig. 8B, C). m^6^A sites mutations also largely abolished LINC02709’s role in promoting liver metastasis (Fig. 8D). To further determine whether LINC02709’ effects depend on the m^6^A/FXR1/TET1 axis, we performed sphere formation assays using LINC02709-overexpressing SNU-398 cells with FXR1 or TET1 depletion. The results revealed that depletion of either FXR1 or TET1 largely abolished LINC02709’s effect on HCC cell stemness properties (Supplementary Fig. S4A, B). In vitro phagocytosis assays demonstrated that depletion of either FXR1 or TET1 largely abolished LINC02709’s effect on KC phagocytosis (Supplementary Fig. S4C, D). In vivo liver metastasis assays showed that depletion of either FXR1 or TET1 also largely abolished LINC02709’s role in promoting liver metastasis (Supplementary Fig. S4E, F). Together, these results strongly support the functional significance of the m^6^A -modified LINC02709/FXR1/TET1/CD47/CD24 regulatory axis in HCC stemness properties and KC phagocytosis, both contributing to HCC liver metastasis.

## Discussion

Intrahepatic metastasis occurs more frequently than extrahepatic spread in HCC and represents a major contributor to tumor recurrence and patient mortality [4]. The identification of molecular drivers underlying HCC intrahepatic metastasis could reveal promising therapeutic targets [54]. LINC02709 (also designated RP11-540A21.2 or AC026250.1) has been previously implicated in the prognosis of both lung adenocarcinoma and HCC [55–58]. Our current findings extend these observations by demonstrating a significant association between LINC02709 expression and HCC intrahepatic metastasis as well as disease recurrence. More importantly, we establish that LINC02709 functionally promotes intrahepatic metastasis, moving beyond its previously recognized role as a prognostic biomarker.

Increasing evidences confirm that CSCs exert critical roles in tumor metastasis [59]. CSCs are also revealed to be responsible for HCC intrahepatic metastasis [6]. Finding new crucial molecules that modulate the stemness properties of HCC cells will provide novel targets for eliminating CSCs and repressing HCC metastasis. Here, we identified LINC02709 as a stemness-associated molecule in HCC. More importantly, LINC02709 functionally enhanced the stemness properties of HCC cells. We further found that the stem cell marker CD24 was upregulated by LINC02709. Functional rescue assays confirmed that CD24 is the critical mediator of the roles of LINC02709 in enhancing stemness properties of HCC cells.

As liver resident macrophage, Kupffer cells (KCs) play a pivotal role in phagocytosing and eliminating invading tumor cells and pathogens [60–62]. However, the molecular regulators of KC phagocytosis remain poorly characterized [63]. While the Kupffer cell lineage-determining factor ID3 was shown to enhance phagocytosis by suppressing *SIRPA* expression [19], other modulators of this process are not well understood. The CD47–SIRPα axis is a well-established anti-phagocytic (“don’t eat me”) signal, where CD47 on tumor cells binds SIRPα (encoded by *SIRPA*) on macrophages to inhibit phagocytosis [64, 65]. Additional pro- and anti-phagocytic signals, including CD24, PD-L1, SLAMF7, and β2-microglobulin [66–69], have been implicated in macrophage function, but their roles in KC-specific phagocytosis remain unclear. In this study, we identified LINC02709 as a novel suppressor of KC phagocytosis, contributing to its pro-metastatic role in HCC. We further demonstrated that CD47 and CD24, expressed on HCC cells, function as anti-phagocytic signals for KCs. LINC02709 suppresses phagocytosis by upregulating CD47 and CD24 in HCC cells. Blockade assays confirmed that CD47 and CD24 upregulation mediates the anti-phagocytic effects of LINC02709.

**Fig. 9:**
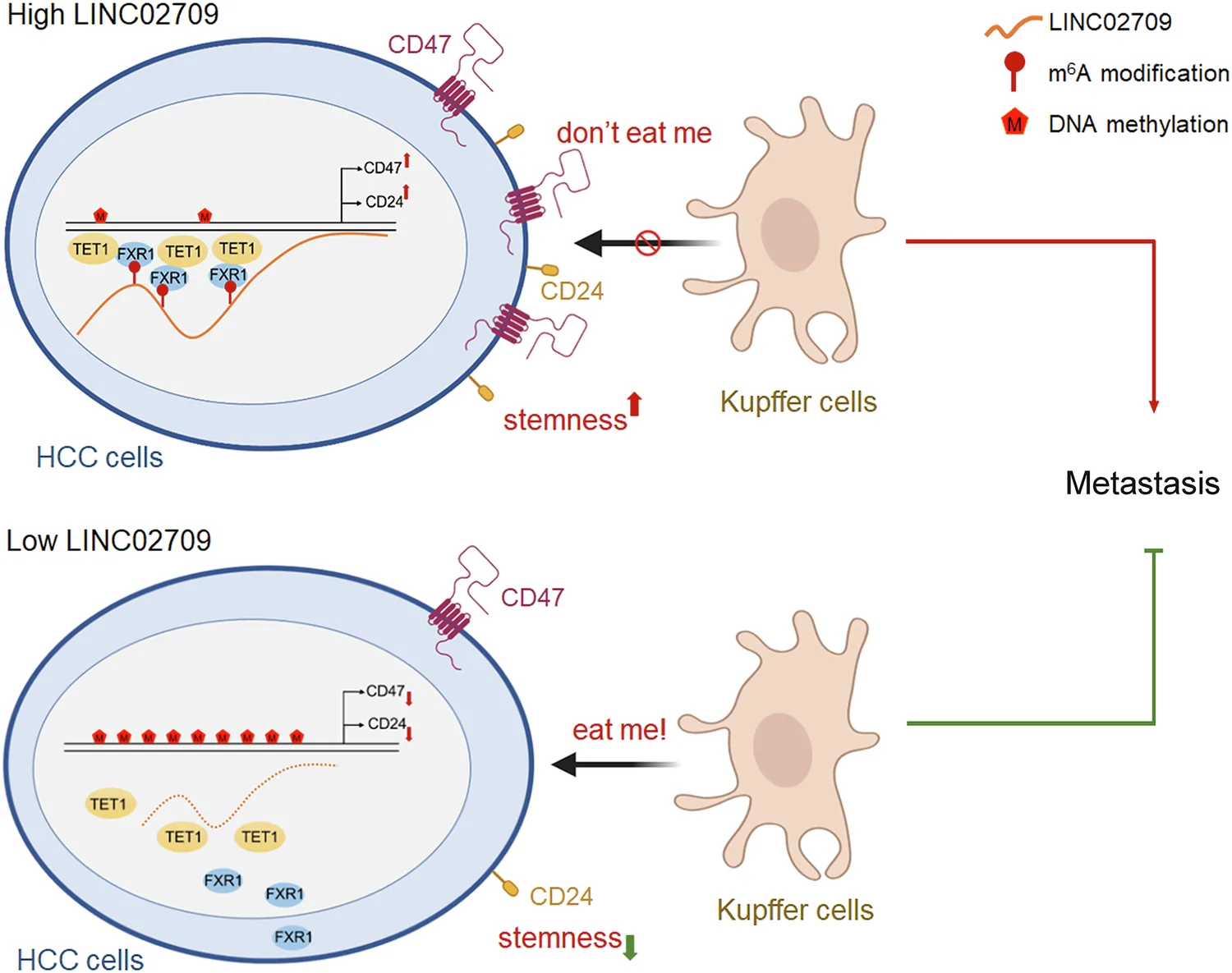
Proposed mechanism of the m^6^A-modified LINC02709/FXR1/TET1/DNA methylation/CD47/CD24 axis in regulating the stemness and phagocytosis of HCC cells by Kupffer cells.

Aberrant m^6^A modification has been linked to various diseases [70–72]. Multiple studies, including ours, have reported HCC-associated dysregulation of m^6^A-modified transcripts such as ZNNT1, FAM111A-DT, RAB11B-AS1, and pri-miR126 [73–76]. While m^6^A modification is known to regulate RNA processing—including splicing, translation, stability, and degradation—via interactions with m^6^A readers [77–79], its role in modulating DNA methylation and histone modifications through m^6^A reader-epigenetic enzyme complexes remains less explored [80]. Our study provides novel insights into the functional consequences of m^6^A modification. We identified three m^6^A modification sites on LINC02709 and demonstrated that its ability to regulate *CD24*, *CD47*, HCC cells stemness properties, KC phagocytosis, and HCC intrahepatic metastasis is strictly m^6^A-dependent. Mechanistically, m^6^A-modified LINC02709 binds the m^6^A reader FXR1 and the DNA demethylase TET1. Furthermore, LINC02709 directly associates with the *CD24*/*CD47* promoters. Through this interaction, m^6^A-modified LINC02709 recruits FXR1-TET1 to the *CD24*/*CD47* promoters, inducing DNA demethylation and transcriptional activation of *CD24* and *CD47*. The upregulated CD24 and CD47 then enhance HCC cells stemness properties and transmit anti-phagocytic signals to KCs, leading to increased metastasis (Fig. 9). Targeting m^6^A-modified LINC02709 using antisense oligonucleotides or small molecular inhibitors represent potential therapeutic strategies for HCC.

In summary, our study identifies LINC02709 as a novel m^6^A-modified transcript that promotes HCC intrahepatic metastasis by enhancing HCC cells stemness properties and suppressing Kupffer cell phagocytosis via epigenetic upregulation of CD24 and CD47. Mechanistically, m^6^A-modified LINC02709 recruits the FXR1/TET1 complex to the *CD24*/*CD47* promoters, inducing DNA demethylation and transcriptional activation. These findings establish the LINC02709/FXR1/TET1/CD24/CD47 axis as a promising therapeutic target for HCC.

There are some limitations in the current study. Using Basic Local Alignment Search Tool (BLAST), we predicted putative LINC02709 complementary binding loci within *CD24*/*CD47*/*CD274*/*ERMAP* promoters. However, our experiments only found the binding of LINC02709 to the *CD47*/*CD24* promoters, and while no binding of LINC02709 to the *CD274*/*ERMAP* promoters. These findings implied that the spatial structures of RNA and DNA also influence their interaction. The specificity of LINC02709’s binding to the *CD47*/*CD24* promoters need further investigation. LINC02709 has no mouse homolog molecule, so we overexpressed and depleted it in human HCC cells. Additionally, healthy human KCs are difficult to obtain, so we measured the phagocytosis of human HCC cells by mouse KCs. Further experiments using human KCs to phagocytose human HCC cells would strengthen the study’s conclusions.

## Materials and methods

### Clinical tissue samples

Eighty-three pairs of HCC tissues and matched adjacent liver tissues were collected from the Affiliated Hospital of Youjiang Medical University for Nationalities with informed consent. The use of tissue samples complied with the Declaration of Helsinki and was approved by the Affiliated Hospital of Youjiang Medical University for Nationalities Institutional Review Board (approval no. YYFY-LL-2023-127). For bioinformatics analysis, TCGA LIHC data were analyzed using the online tools GEPIA (Gene Expression Profiling Interactive Analysis, http://gepia.cancer-pku.cn/index.html) and R2 Genomics Analysis and Visualization Platform (https://hgserver1.amc.nl/cgi-bin/r2/main.cgi).

### Cell lines and culture

Human HCC cell lines SK-HEP-1 (SCSP-5281), SNU-398 (SCSP-5206), Hep3B (SCSP-5045), HuH-7 (SCSP-526), and HCCLM3 (SCSP-5093) were purchased from the National Collection of Authenticated Cell Cultures (Shanghai, China). The immortalized normal liver cell line THLE-2 (CRL-2706) was obtained from the American Type Culture Collection (Manassas, VA, USA). SK-HEP-1 cells were cultured in Minimum Essential Medium (MEM; Gibco, Carlsbad, CA, USA) supplemented with 10% fetal bovine serum (FBS; Gibco) and 1% non-essential amino acids (NEAA; Gibco). SNU-398 cells were cultured in RPMI 1640 Medium (Gibco) with 10% FBS. Hep3B cells were cultured in MEM (Gibco) with 10% FBS, 1% NEAA, 1% GlutaMAX^TM^ (Gibco), and 1% Sodium Pyruvate (Gibco). HuH-7 cells were cultured in Dulbecco’s Modified Eagle Medium (DMEM; Gibco) with 10% FBS, 1% GlutaMAX, and 1% Sodium Pyruvate. HCCLM3 cells were cultured in DMEM (Gibco) with 10% FBS. THLE-2 cells were cultured with the BEGM Bullet Kit (Lonza, Basel, Switzerland). All cells were maintained at 37 °C in a humidified 5% CO_2_ atmosphere. The cell lines were authenticated by short-tandem-repeat DNA profiling and routinely tested as mycoplasma-free.

### RNA extraction, reverse transcription, and real-time quantitative polymerase chain reaction (qPCR)

Total RNA was isolated from tissues and cells using RNA isolater Total RNA Extraction Reagent (Vazyme, Nanjing, China). Subcellular RNA fractionation was conducted as previously described [49], and RNA from each fraction was extracted using TRIzol^TM^ Reagent (Invitrogen, Carlsbad, CA, USA). Reverse transcription was performed using HiScript III RT SuperMix for qPCR (Vazyme). The resulting cDNA was then subjected to real-time qPCR using ChamQ Universal SYBR qPCR Master Mix (Vazyme). β-actin served as the internal reference. The primer sequences for qPCR are listed in Supplementary Table S1.

### Vectors and stable cell lines

The LINC02709 sequence was PCR-amplified using Fast Pfu DNA Polymerase (Novoprotein, Suzhou, China) and cloned into the *Eco*R I and *Not* I sites of pcDNA^TM^3.1(+) vector (Invitrogen) with the NovoRec plus One step PCR Cloning Kit (Novoprotein) to construct the LINC02709 expression vector. The m^6^A sites-mutated LINC02709 expression vector was generated using the Fast Mutagenesis System (TransGen, Beijing, China). Primer sequences for vectors construction are listed in Supplementary Table S1. Two pairs of cDNA oligonucleotides targeting LINC02709 were cloned into the lentiviral shRNA expressing vector (LV3) (GenePharma, Shanghai, China) to produce LINC02709-knockdown lentiviruses. The shRNA sequences are provided in Supplementary Table S1. shRNA clones targeting CD24, CD47, FXR1, or TET1 were purchased from FulenGen (Guangzhou, China).

To construct HCC cells stably expressing wild-type or m^6^A sites-mutated LINC02709, wild-type or m^6^A sites-mutated LINC02709 expression vectors were transfected into SK-HEP-1 and SNU-398 cells using GP-transfect-Mate (GenePharma), followed by selection with 800 µg/mL neomycin (InvivoGen, San Diego, CA, USA) for 4 weeks. To generate LINC02709 stable knockdown HCC cells, SNU-398 and HCCLM3 cells were infected with LINC02709-knockdown shRNA lentiviruses and subsequently selected with 1 µg/mL puromycin (InvivoGen). To generate SNU-398 cells with LINC02709 overexpression and concurrent CD24, CD47, FXR1, or TET1 knockdown, shRNA clones targeting CD24, CD47, FXR1, or TET1 were transfected into LINC02709-overexpressing SNU-398 cells using GP-transfect-Mate, followed by selection with 800 µg/mL neomycin and 1 µg/mL puromycin.

### Hepatic metastasis assays

Male athymic BALB/c nude mice (6–8 weeks old) were obtained from SiPeiFu Biotechnology (Beijing, China). To assess hepatic metastasis of HCC cells, the indicated HCC cells were intrasplenically inoculated into nude mice which were anesthetized with isoflurane. At the indicated time points, the mice were euthanized using CO_2_ inhalation and the livers were resected and subjected to hematoxylin and eosin (H&E) staining. All experiments were conducted in accordance with international guidelines and the Basel Declaration, with approval from the Affiliated Hospital of Youjiang Medical University for Nationalities Institutional Review Board (approval no. YYFY-LL-2023-127). No statistical method was used to determine sample size. The experiments were not randomized. The investigators recording hepatic metastasis were blinded to mouse allocation. The hepatic metastasis assays were repeated independently.

### Spheroids formation assay

Cell stemness properties were evaluated using spheroid formation assays as we previously described [50]. Briefly, 1000 cells/well were seeded in 6-well ultra-low attachment culture dishes. After incubation for 10 days, the spheroids were photographed and measured using an inverted microscope.

### In vitro phagocytosis assays

F4/80^+^ KCs were isolated from the liver as previously described [18]. 0.25 × 10^5^ KCs were co-cultured with 1 × 10^5^ CFSE-labeled HCC cells for 2 h in ultra-low-attachment 96-well U-bottom plates (Corning, New York, USA). After co-culture, cells were stained with an anti-F4/80 antibody (17-4801-82; eBioscience, San Diego, CA, USA). Phagocytosis efficiency was determined by measuring CFSE intensity in KCs using flow cytometry. For CD47 and CD24 neutralization experiments, HCC cells were pre-treated with 10 µg/mL CD47 neutralizing antibody Magrolimab (HY-P99029; MedChemExpress, Shanghai, China) and/or CD24 neutralizing antibody ATG-031 (HY-P99176; MedChemExpress).

### In vivo phagocytosis assays

CFSE-labeled HCC cells were intrasplenically inoculated into nude mice. At 12 h post-inoculation, liver non-parenchymal cells (NPCs) were isolated as previously described [18]. Live NPCs (7-AAD-negative; BD Biosciences, Franklin Lakes, NJ, USA) were stained with the antibodies against CD45.2 (109807; BioLegend, San Diego, CA, USA), CD11b (101215; BioLegend) and F4/80 (123115; BioLegend) to assess phagocytosis efficiency of CFSE^+^ HCC cells by KCs (CD45^+^CD11b^int^F4/80^+^).

### Flow cytometry analysis

Flow cytometry was performed using an Attune NxT Flow Cytometer (Thermo Fisher Scientific, Waltham, MA, USA). For detection of surface expression, cells were stained with the antibodies against CD24 (311105; BioLegend) or CD47 (323123; BioLegend).

### Bisulfite DNA sequencing

Genomic DNA was isolated using the TIANamp Genomic DNA Kit (TIANGEN, Beijing, China) and subsequently treated with sodium bisulfite using the EZ DNA Methylation-Gold^TM^ Kit (Zymo Research, Irvine, CA, USA). Bisulfite-converted DNA was then PCR-amplified with Fast Pfu DNA Polymerase (Novoprotein) using primers listed in Supplementary Table S1. The amplified products were cloned into the pMD-19T Vectors (Takara, Dalian, China) and transformed into *Escherichia coli* DH5α competent cells. Positive clones were selected and subjected to Sanger sequencing for methylation analysis.

### Cleavage under targets & release using nuclease (CUT&RUN)

The genomic binding profiles of DNA methyltransferases and demethylases were assessed using the CUT&RUN Assay Kit (Cell Signaling Technology, Danvers, MA, USA) with the antibodies against DNMT1 (ab320817; Abcam, Cambridge, UK), DNMT3A (32578; Cell Signaling Technology), DNMT3B (57868; Cell Signaling Technology), TET1 (40142; Cell Signaling Technology), TET2 (18950; Cell Signaling Technology), and TET3 (PA5-31860; Invitrogen). Enriched DNA fragments were quantified by qPCR using the primers listed in Supplementary Table S1.

### Chromatin isolation by RNA purification (ChIRP)

RNA-DNA interactions were examined by ChIRP assays using the EZ-Magna ChIRP RNA Interactome Kit (Millipore, Billerica, MA, USA) and antisense DNA probes targeting LINC02709 (sequences in Supplementary Table S1). Enriched DNA was analyzed by qPCR with primers provided in Supplementary Table S1.

### RNA immunoprecipitation (RIP) and Methylated RNA immunoprecipitation (MeRIP) assays

Protein-RNA interactions were analyzed by RIP assays using the EZ-Magna RIP RNA-Binding Protein Immunoprecipitation Kit (Millipore) and primary antibodies against FXR1 (03-176; Millipore) or TET1 (40142; Cell Signaling Technology). Enriched RNA was analyzed by qPCR. m^6^A modification levels were determined by MeRIP assays using the Magna MeRIP m^6^A Kit (Millipore). Immunoprecipitated m^6^A-modified LINC02709 was quantified by qPCR.

### Statistical analysis

Statistical analyses were performed using GraphPad Prism (version 10.0). Differences were assessed by Student’s *t* test, one-way ANOVA with Dunnett’s multiple comparisons test, Mann–Whitney test, Wilcoxon matched-pairs signed rank test, Kruskal–Wallis test with Dunn’s multiple comparisons test, Kaplan–Meier survival analysis, Spearman correlation analysis, or Pearson chi-square tests as indicated in figure and table legends. A *p*-value < 0.05 was considered statistically significant.

## Supplementary information

Supplementary materials

## Data Availability

The datasets generated and/or analyzed during this study are available from the corresponding author on reasonable request.
