# Supplementary material for: LINC02709 facilitates hepatocellular carcinoma metastasis by increasing stem cell-like properties and suppressing Kupffer cell phagocytosis
Source: Cell Death Discov. 2026 Aug 12;12:448. doi: 10.1038/s41420-026-03276-8 (PMC13748585; doi:10.1038/s41420-026-03276-8)
Supplement: Supplementary file 1 [file 41420_2026_3276_MOESM1_ESM.pdf]

Supplementary Figures

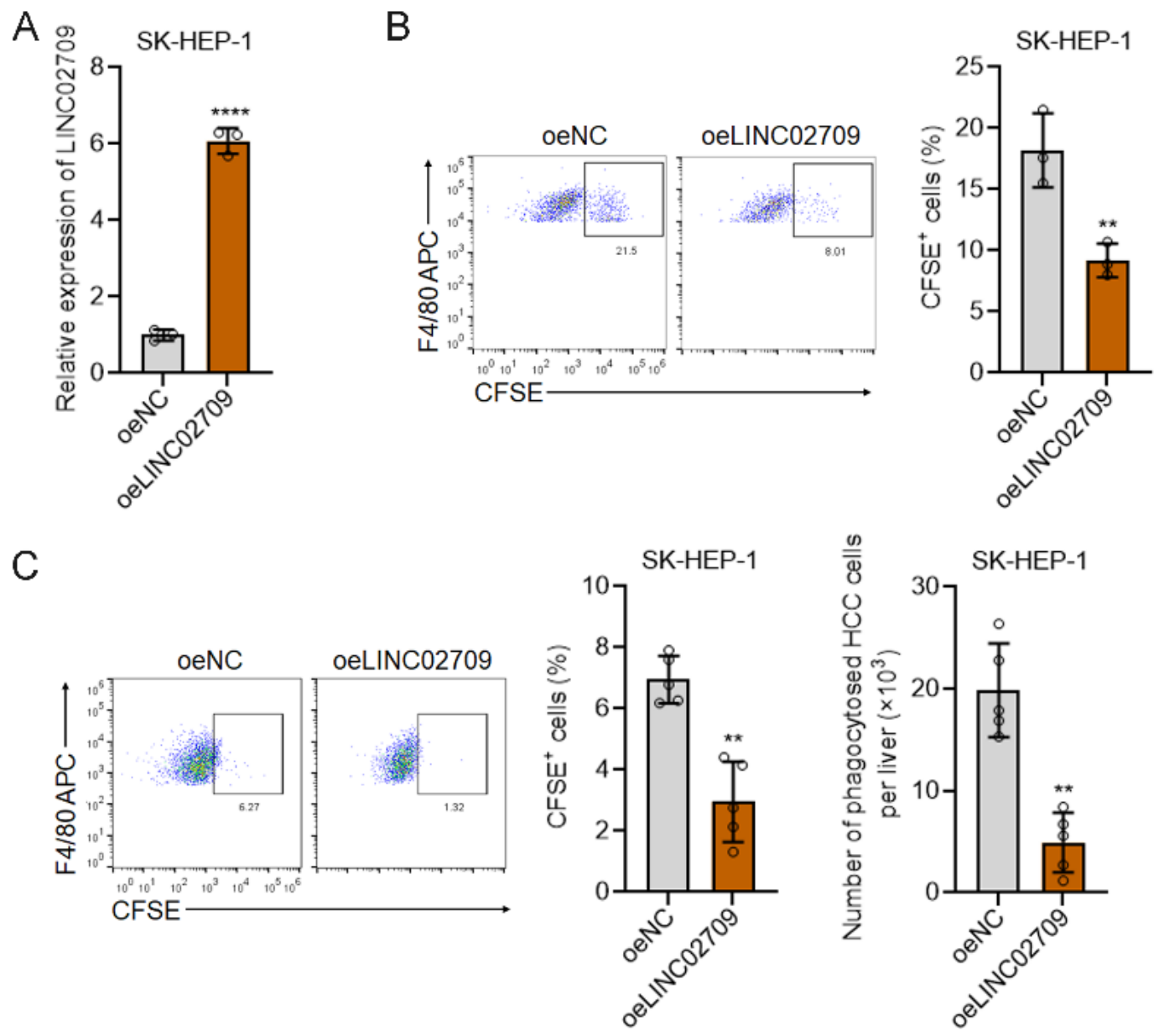

Fig. S1 LINC02709 suppresses the phagocytosis of SK-HEP-1 cells by Kupffer cells. (**A**) qPCR validation of LINC02709 overexpression in SK-HEP-1 cells. (**B**, **C**) Flow cytometry analysis of phagocytosis of CFSE-labeled SK-HEP-1 cells by KCs *in vitro* (**B**) and *in vivo* (**C**, 12 h post-intrasplenic inoculation). Data are presented as mean ± SD of n = 3 biological replicates (**A**, **B**) or n = 5 mice per group (**C**). **p < 0.01, ****p < 0.0001 by Student's *t*-test (**A**, **B**) or Mann-Whitney test (**C**).

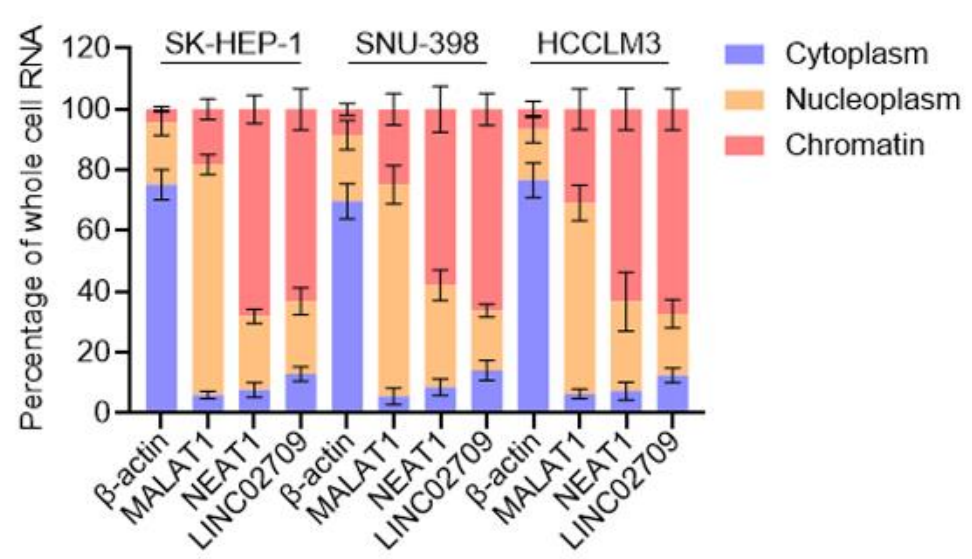

Fig. S2 Subcellular localization of LINC02709 in fractionated HCC cells. β-actin

(cytoplasm), MALAT1 (nucleoplasm), and NEAT1 (chromatin) were used as controls. Data are presented as mean ± SD of n = 3 biological replicates.

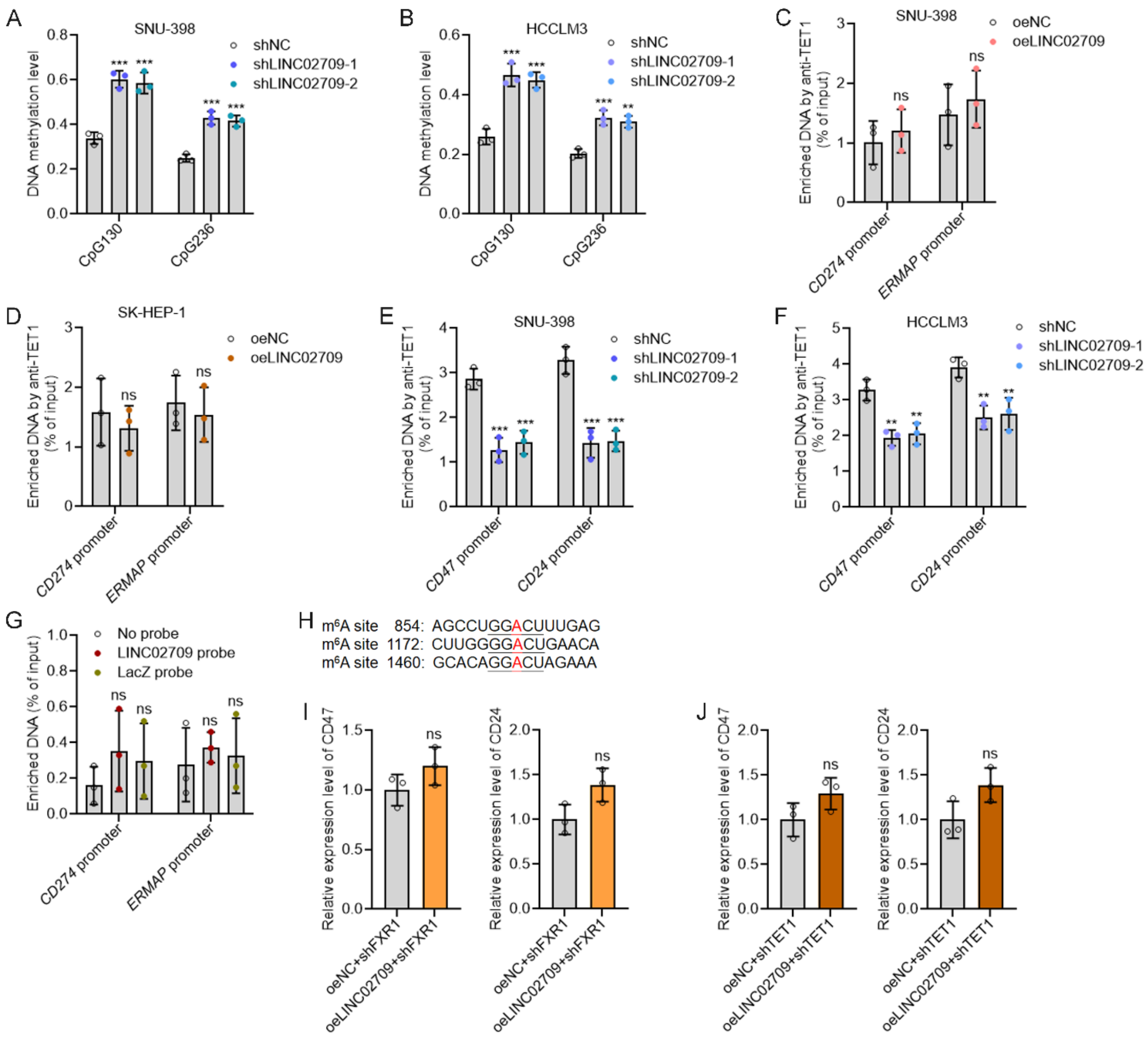

Fig. S3 LINC02709 knockdown increases DNA methylation levels at *CD47*/*CD24* promoters through reducing the binding of TET1 to *CD47*/*CD24* promoters. (**A**, **B**) Bisulfate sequencing of *CD47*/*CD24* promoter methylation in LINC02709-knockdown SNU-398 (**A**) or HCCLM3 (**B**) cells. (**C**, **D**) CUT&RUN analysis of TET1 binding to *CD274*/*ERMAP* promoters in SNU-398 (**C**) or SK-HEP-1 (**D**) cells with LINC02709 overexpression. (**E**, **F**) CUT&RUN analysis of TET1 binding to

CD47/*CD24* promoters in LINC02709-knockdown SNU-398 (**E**) or HCCLM3 (**F**) cells. (**G**) ChIRP analysis of the binding of LINC02709 to *CD274*/*ERMAP* promoters in SNU-398 cells. LacZ probe served as negative control probe. (**H**) Predicted m^6A modification sites on LINC02709. (**I**) qPCR analysis of CD47 and CD24 mRNA levels in SNU-398 cells with LINC02709 overexpression and FXR1 knockdown. (**J**) qPCR analysis of CD47 and CD24 mRNA levels in SK-HEP-1 cells with LINC02709 overexpression and TET1 knockdown. Data are presented as mean ± SD of n = 3 biological replicates. **p < 0.01, ***p < 0.001, ns, not significant, by one-way ANOVA with Dunnett's multiple comparisons test (**A**, **B**, **E**-**G**) or Student’s *t*-test (**C**, **D**, **I**, **J**).

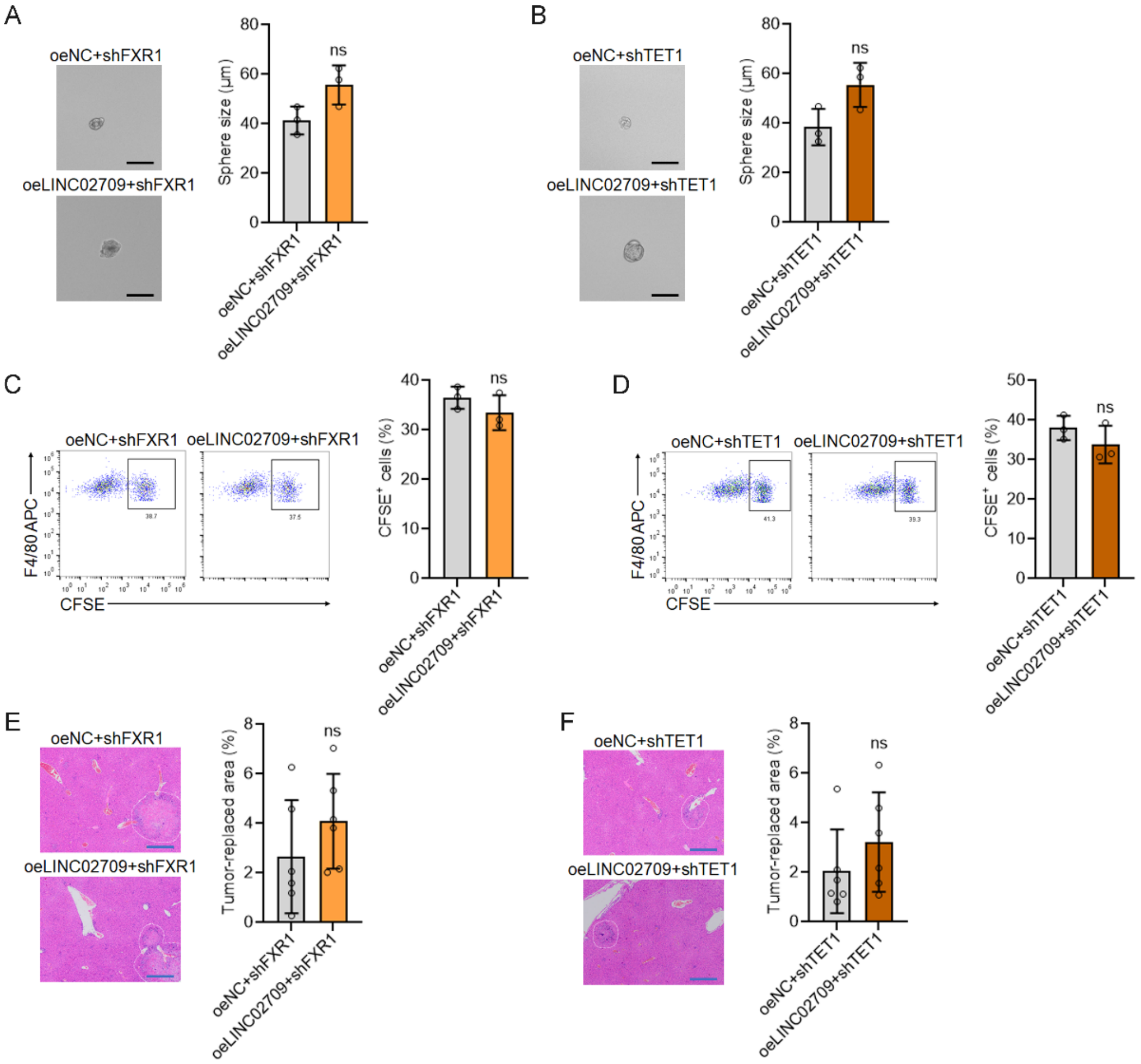

Fig. S4 FXR1 and TET1 mediate LINC02709' effects on HCC stemness property and Kupffer cell phagocytosis. (**A**) The spheroids form ability of SNU-398 cells with LINC02709 overexpression and FXR1 knockdown. Scale bars = 100 μm. (**A**) The spheroids form ability of SNU-398 cells with LINC02709 overexpression and TET1 knockdown. Scale bars = 100 μm. (**C**) Flow cytometry analysis of *in vitro* phagocytosis of CFSE-labeled SNU-398 cells with LINC02709 overexpression and FXR1 knockdown by KCs. (**D**) Flow cytometry analysis of *in vitro* phagocytosis of CFSE-labeled SNU-398 cells with LINC02709 overexpression and TET1 knockdown by KCs. (**E**) Liver metastasis assessed by H&E staining at day 49 after intrasplenic injection of 5 × 10^6 SNU-398 cells with LINC02709 overexpression and FXR1

knockdown. Scale bars = 500 μm. (**F**) Liver metastasis assessed by H&E staining at day 49 after intrasplenic injection of 5 × 10^6 SNU-398 cells with LINC02709 overexpression and TET1 knockdown. Scale bars = 500 μm. Data are presented as mean ± SD of n = 3 biological replicates (**A**-**D**) or n = 6 mice per group (**E**, **F**). ns, not significant, by Student's *t*-test (**A**-**D**) or Mann-Whitney test (**E**, **F**).

Table S1. Primer, shRNA, and probe sequences used in this study.

**Primers for qPCR**		**Sequence (5'-3')**
LINC02709	Forward	GAGATTATGGCGAAGCATTTTA
	Reverse	TTCAAGGCTACTACTGGAGAG
m^6A sites mutated LINC02709	Forward	CTGCGGGTCTTGGGGTCT
	Reverse	CCCTGACCCTTTCTAGACCTG
β-actin	Forward	GGGAAATCGTGCGTGACATTAAG
	Reverse	TGTGTTGGCGTACAGGTCTTTG
MALAT1	Forward	TGGAGAAATAGTAGATGGC
	Reverse	GGTGAGGAAGTAAAAACAG
NEAT1	Forward	GTCTTTCCATCCACTCACGTCT
	Reverse	GGACAACTAGATGCCGAGGTAG
CD47	Forward	TTGTGACTTCTACAGGGAT
	Reverse	CAGCGAGGATATAGGCTA
CD274	Forward	CTGACATTCATCTTCCGTTTA
	Reverse	CTTTCGCCAGGTTCCATTT
CD24	Forward	TGCTGGCACTGCTCCTAC
	Reverse	CGAAGAGACTGGCTGTTGAC
ERMAP	Forward	TCATCATGGTGTGCCTTTGCC
	Reverse	CTCCGGAGTTTCTTGACAGCT
SLAMF7	Forward	CACTGTGGAAATACCGAAAA
	Reverse	GAGCAGAGACTTAGGGGAG
B2M	Forward	GGCTATCCAGCGTACTCCA
	Reverse	ACGGCAGGCATACTCATCT
**Primers for vector construction**		

LINC02709 expression	Forward	AGTCCAGTGTGGTGGAATTCTCTCCACTCCTGCCTCCTG
	Reverse	CCTCTAGACTCGAGCGGCCGCGAGAATAGACAATTGTATTTCCAG
LINC02709 mutation (854 site)	Forward	CTCTCCTATCAGCCTGGTCTTTGAGGGG
	Reverse	ACCAGGCTGATAGGAGAGCAGCGTTACTG
LINC02709 mutation (1172 site)	Forward	CTGCGGGTCTTGGGGTCTGAACAGAATGG
	Reverse	ACCCCAAGACCCGCAGCCCACTGCCC
LINC02709 mutation (1160 site)	Forward	GGAAGCTGAGCACAGGTCTAGAAAGGG
	Reverse	ACCTGTGCTCAGCTTCCATTGAGTCTTC
LINC02709 shRNA-1	Forward	GATCCGGTTGCCTCTCTCTGTAATATTTCAAGAGAATATTACAGAGAGAGGCAACCTTTTTTG
	Reverse	AATTCAAAAAAGGTTGCCTCTCTCTGTAATATTCTCTTGAAATATTACAGAGAGAGGCAACCG
LINC02709 shRNA-2	Forward	GATCCGCATCACAGCGAGACTTCTTATTCAAGAGATAAGAAGTCTCGCTGTGATGCTTTTTTG
	Reverse	AATTCAAAAAAGCATCACAGCGAGACTTCTTATCTCTTGAATAAGAAGTCTCGCTGTGATGCG
shRNA-NC	Forward	GATCCGTTCTCCGAACGTGTCACGTTTCAAGAGAACGTGACACGTTCGGAGAACTTTTTTG
	Reverse	AATTCAAAAAAGTTCTCCGAACGTGTCACGTTCTCTTGAAACGTGACACGTTCGGAGAACG
**PCR primers for DNA methylation detection**		
CD47 promoter (CpG130)	Forward	TTGGGTTTTATTGTTTTTTT
	Reverse	AATTAACCCTAATCAAAAAATA
CD24 promoter (CpG236)	Forward	GTTTTGTTTTTAGGTTTTATT
	Reverse	CTCAAAATACTCCACCAATA
**antisense DNA probes against LINC02709 for ChIRP**		
1		GAGGGGTGATGTAGAGTCAA
2		TGAGAAGATCTGGCAGGTGA
3		GTTTTGTCTTCATGCATGTT
4		CAGGCCTTTAGGGCACTAAG
5		ACTGCAGTTAGCTGGAAGAT
6		TGATAGGAGAGCAGCGTTAC
7		ATGCATGGGACCAGGACGAT
8		GACAACACTTTCTGCTTGGA
9		AAACCCCATTCTGTTCAGTC

10		GAATGCAGGCCTTTGTGTTT
11		TACATCTGTGGTGGACACTA
12		CACCCCTACATACAGAGGAG
13		CTTCTAGGATGCTGTTCATT
14		CTCTAGAAGCCTGAACTGGC
**qPCR primers for CUT&RUN and ChIRP**		
CD47 promoter	Forward	AGGAGGCTGAACCCTGTGT
	Reverse	GGTGGGAGTGAAAGCAAAG
CD24 promoter	Forward	CTGTTGCCCGTCATTCGTTT
	Reverse	CCTCTTTGTGCCGGTTCATT
ACTB promoter	Forward	GCCGACTTCAGAGCAACTG
	Reverse	TGACTCCCCCAACACCAC
CD274 promoter	Forward	CCTTGAAGAGGCTTTTATC
	Reverse	GAACGCACCTTGATTTTAC
ERMAP promoter	Forward	GGCAAGTCTCCTCCCCACT
	Reverse	CATTTCACCCGCACACAGC
